# Using Smartphones and Health Apps to Change and Manage Health Behaviors: A Population-Based Survey

**DOI:** 10.2196/jmir.6838

**Published:** 2017-04-05

**Authors:** Clemens Ernsting, Stephan U Dombrowski, Monika Oedekoven, Julie L O´Sullivan, Melanie Kanzler, Adelheid Kuhlmey, Paul Gellert

**Affiliations:** ^1^ Institute of Medical Sociology Charité - Universitätsmedizin Berlin Berlin Germany; ^2^ Faculty of Natural Sciences University of Stirling Stirling United Kingdom; ^3^ Pfizer Deutschland GmbH Berlin Germany

**Keywords:** telemedicine, eHealth, mHealth, smartphone, mobile apps, health promotion, chronic disease, health literacy, quality of life

## Abstract

**Background:**

Chronic conditions are an increasing challenge for individuals and the health care system. Smartphones and health apps are potentially promising tools to change health-related behaviors and manage chronic conditions.

**Objective:**

The aim of this study was to explore (1) the extent of smartphone and health app use, (2) sociodemographic, medical, and behavioral correlates of smartphone and health app use, and (3) associations of the use of apps and app characteristics with actual health behaviors.

**Methods:**

A population-based survey (N=4144) among Germans, aged 35 years and older, was conducted. Sociodemographics, presence of chronic conditions, health behaviors, quality of life, and health literacy, as well as the use of the Internet, smartphone, and health apps were assessed by questionnaire at home visit. Binary logistic regression models were applied.

**Results:**

It was found that 61.25% (2538/4144) of participants used a smartphone. Compared with nonusers, smartphone users were younger, did more research on the Internet, were more likely to work full-time and more likely to have a university degree, engaged more in physical activity, and less in low fat diet, and had a higher health-related quality of life and health literacy. Among smartphone users, 20.53% (521/2538) used health apps. App users were younger, less likely to be native German speakers, did more research on the Internet, were more likely to report chronic conditions, engaged more in physical activity, and low fat diet, and were more health literate compared with nonusers who had a smartphone. Health apps focused on smoking cessation (232/521, 44.5%), healthy diet (201/521, 38.6%), and weight loss (121/521, 23.2%). The most common app characteristics were planning (264/521, 50.7%), reminding (188/521, 36.1%), prompting motivation (179/521 34.4%), and the provision of information (175/521, 33.6%). Significant associations were found between planning and the health behavior physical activity, between feedback or monitoring and physical activity, and between feedback or monitoring and adherence to doctor’s advice.

**Conclusions:**

Although there were many smartphone and health app users, a substantial proportion of the population was not engaged. Findings suggest age-related, socioeconomic-related, literacy-related, and health-related disparities in the use of mobile technologies. Health app use may reflect a user’s motivation to change or maintain health behaviors. App developers and researchers should take account of the needs of older people, people with low health literacy, and chronic conditions.

## Introduction

### Mobile Health Apps

An increasing number of people take advantage of smartphones for health issues. Mobile health apps have risen in popularity, providing new opportunities to change health-related behaviors and to manage chronic conditions [1]. Typical health apps provide immediate access to health information, medication reminders, or help track progress in physical exercise regime. However, the factors related with smartphone and health app use are not yet fully understood. This study investigated the associations between demographic and health-related factors, common chronic conditions, and health behaviors with smartphone and health app use.

Research has shown associations between (1) health app use and behavior, and (2) app use and management of chronic conditions [2-6]. Krebs and Duncan [7] examined the health app use among mobile phone users in the United States. Their findings suggest an association between health app use and sociodemographic factors; that is, app users were younger, had a higher income, were better educated, were more likely to be Hispanic, and had a higher body mass index (BMI). A systematic review of mobile health interventions based primarily on randomized controlled trials by Riley et al [2], who investigated the effectiveness and theoretical background of mobile interventions such as apps for smartphones or tablets. Results demonstrated that intervention participants were more successful in changing a variety of health behaviors and behavior-related outcomes, including physical activity, smoking cessation, healthy diet, weight loss, medication adherence, improvement of blood pressure control, and improvement of blood sugar control [2].

### Factors Related to the Use of Health Apps

In a cross-sectional survey, Cho et al [8] examined factors related to the use of health apps and found a significant correlation between app use and health consciousness as well as an indirect link between health literacy and health-app use efficacy on health app use. Bailey et al [9] found widespread age and health literacy-related disparities in technology access, with older and less literate individuals being less likely to own smartphones and use the Internet, especially for health reasons. In a recent longitudinal survey, Levine et al [10] found that older people (mean age 75 years) used digital health at low rates but there were modest increases from 2011 to 2014. Despite a growing body of research on health apps, there is still a lack of evidence concerning the associations of sociodemographic, medical, and behavioral factors such as health-related quality of life, health behaviors, Internet use, multiple chronic conditions, and health app use.

### Health App Characteristics

In addition to individual factors, health app characteristics such as monitoring progress or reminding may be related to the actual health behavior of the users. Findings from a systematic review that analyzed characteristics of Internet-based interventions revealed that theory-based approaches were associated with increases in effect sizes [11]. In a recent content analysis, Morrissey et al [12] examined the extent to which certain established behavior change techniques were used in apps designed to promote medication adherence. They found a range from 0 to 7 behavior change techniques implemented in these apps. Planning, prompts or cues, monitoring, and feedback on behavior were included most frequently.

### Aims of the Study

The aims of this study were to (1) investigate the prevalence of smartphone and health app use, (2) identify sociodemographic, medical, and behavioral correlates of smartphone and health app use, and (3) explore the correlations between behaviors targeted by the apps and actual health behavior, and health app characteristics and actual health behavior.

## Methods

### Sample and Procedure

A population-based sample of 4144 individuals from Germany participated in this cross-sectional survey. Data were collected in July 2015. An external agency was authorized to run the study. The agency employed interviewers to recruit participants on their own responsibility. To enhance the representativeness of the study, interviewers got specification concerning the composition of the sample, that is, the sample was stratified for sex, age, German federal state, and education to increase representativeness. Participants had to meet the following inclusion criteria: (1) German resident, (2) sufficient German language skills, (3) aged 35 years and older. There were no exclusion criteria. After a first contact, appointments for home visits were made with a response rate of 55%. Computer-assisted personal interviews (CAPI) were conducted by external, trained interviewers at home visits. Of the interviewed individuals, 7% refused to finish the survey and their data were subsequently deleted. The mean time participants need to finish the survey was 29 min. This study was conducted in compliance with the Declaration of Helsinki; written informed consent was obtained from participants [13].

### Measures

#### Sociodemographics

Sex, age, height, weight, education (International Standard Classification of Education, ISCED) [14], occupation, income, and first language were assessed by standard survey items. Post-tax household income by month was categorized: low <€2100; moderate €2100-3600; high >€3600 (1 Euro=1.1 US dollar [October 6, 2016]).

#### Chronic Conditions

Chronic conditions were assessed by asking participants: “Do you suffer from one or more of the following chronic conditions: (1) cardiovascular disease, (2) cancer, (3) respiratory diseases, (4) diseases of the musculoskeletal system, (5) major depression, (6) chronic pain, (7) diabetes, (8) hypertension, and (9) other chronic condition.” Furthermore, BMI was calculated by using self-reported weight and height (BMI=weight (in kg)/height (in squared meter). A BMI of 30 or above was considered obese, in accordance with the World Health Organization (WHO) definition [15]. Chronic conditions were summed up to the variable *multiple chronic conditions* ranging from “none,” “one,” “two,” to “three or more.”

#### Health Behaviors

Health behaviors were assessed by providing a list of common health-related behaviors (ie, smoking, physical activity, balanced diet, low fat diet, and adherence to doctor’s advice). These items were derived from the German Aging Survey (2013) and were adjusted for our survey. *Adherence to doctor’s advice* was taken from the 16-item short-form of the European Health Literacy Survey Questionnaire (HLS-EU-Q) instrument [16]. For *smoking*, participants were asked: “Do you smoke on a daily basis?” To assess *physical activity*, participants were asked: “Are you regularly physically active (following WHO recommendation, ie, 30 min of moderate activity at least 5 times per week or 30 min of intensive activity at least 3 times per week [17])?” *Balanced diet* was measured by asking participants: “Do you follow a balanced diet, that is, eat fruits and vegetables with every meal and including many wholegrain products?” *Low fat diet* was assessed by asking participants: “Do you follow a low fat diet, that is, eat few animal fats, peanuts, potato chips, and convenience food?” *Adherence to doctor’s advice* was assessed by asking participants: “On a scale from very easy to very difficult, how easy would you say it is to follow physician’s and pharmacist’s instructions?” The answer had a 4-point response format on a Likert scale. The answers “very easy” and “easy” were defined as adherence (coded 1=present) and the answers “difficult” and “very difficult” were defined as problematic adherence (coded 0=absent).

#### Health-Related Quality of Life and Perceived Health Literacy

*Health-related quality of life* was assessed by the European Health Interview Survey-Quality of Life (EUROHIS-QOL) 8-item index [18] with a Cronbach alpha of .90 in this analysis. Example items included: “How would you rate your quality of life?” and “How satisfied are you with your health?” All answers were given on a 5-point Likert scale. *Health literacy* was assessed by the 16-item short-form of the HLS-EU-Q instrument with a Cronbach alpha of .87 and a range from 0 to 50 indicating the perceived capability of an individual to acquire, understand, and act on health information [19]. An example item was: “On a scale from very easy to very difficult, how easy would you say it is to find information on treatments of illnesses that concern you?” Answers had a 4-point response format on a Likert scale.

#### Internet Use

Internet use was assessed by asking participants: “Do you use the Internet for obtaining information about health conditions or other health-related issues?” (coded as 1=present, 0=absent).

#### Smartphone and App Use

Smartphone use was assessed by asking participants: “Do you own a smartphone, that is, an Internet-compatible cell phone?” (coded 1=present, 0=absent).

*Health app use* and *behaviors targeted by the apps* were measured by asking participants: “Think about the last 12 months. Did you use smartphone apps to improve one of the following behaviors?: (1) to quit smoking, (2) to be regularly physically active, (3) to maintain a healthy diet, (4) to reduce weight, (5) to take medications regularly, (6) to improve blood pressure control, (7) to improve blood sugar control, and (8) I do not use smartphone apps to improve behaviors.” The health behaviors and behavior-related outcomes targeted by the apps were based on findings from a systematic review of health apps [2]. People who chose one or more of these behaviors were classified as health app users (coded 1=present) and people who chose “I do not use smartphone apps to improve behaviors” were classified as nonusers (coded 0=absent). *Behaviors targeted by the apps* were coded 1=present, 0=absent. There was no option to name alternative behaviors targeted by the apps.

*App characteristics* were assessed by asking participants: “Did the health apps you used contain one of the following characteristics: (1) provision of information on the target behavior, (2) motivational messages, (3) goal setting and action planning, (4) reminder, (5) information on the current status and individual progress, and (6) I do not use any of these app characteristics.” App characteristics were chosen in accordance to the behavior change technique taxonomy by Abraham and Michie [20], that is, *providing information, planning, reminding, providing feedback or monitoring*. Furthermore, the additional app characteristic *prompting motivation* was derived from a systematic review of mobile health interventions conducted by Riley et al [2]. It refers to the mobile intervention *messages containing motivational support* reviewed by Riley and encompasses the behavior change techniques such as the *provision of general encouragement* or *provision of contingent rewards* as outlined by Abraham. Participants were asked to choose one or more of the characteristics, coded 1=present and 0=absent. There was no option to name alternative app characteristics.

### Statistical Analyses

We analyzed the entire sample as well as specific subgroups. Beyond smartphone users and nonusers, we compared health app users and non-health app users among the smartphone users. Binary logistic regressions with smartphone use (n=4144) and health app use (n=2538; subsample of smartphone users) as outcomes were conducted. Covariates were sex, age, number of chronic conditions, perceived health literacy, health-related quality of life, and Internet use. Other covariates were the health behaviors smoking, physical activity, balanced diet, and low fat diet.

Furthermore, we explored correlations between behaviors targeted by the apps and app characteristics with reported behavior by applying binary logistic regression models. We ran separate analyses for each behavior, such as for smoking, physical activity, balanced diet, low fat diet, and adherence to doctor’s advice. Health behaviors and behavior-related outcomes targeted by the apps were regular physical activity, smoking cessation, healthy diet, weight loss, medication adherence, improving blood pressure control, and improving blood sugar control. App characteristics comprised providing information, prompting motivation, planning, reminding, and feedback or monitoring. Sex, age, behaviors targeted by the apps, and app characteristics were included as covariates into the analyses.

Additionally, we applied Hochberg’s multistep procedure which is a slightly less restrictive alternative to the Bonferroni approach to correct for multiplicity (Hochberg [21]; for a discussion, see Streiner [22]).

## Results

### Characterization of the Sample

A total of 4144 individuals completed the population-based nationwide survey (see Table 1). The mean age was 57 years (SD 13.5) and 50.96% (2112/4144) were women. It was found that 68.97% (2858/4144) had a vocational qualification, 18.15% (752/4144) had a university degree, and 12.89% (534/4144) had no or basic qualification. Most participants were working full-time (2224/4144, 53.67%) and had a medium household income (1930/4144, 46.57%).

The majority of the sample (2231/4144, 53.84%) reported no chronic conditions, whereas 30.84% (1278/4144) reported having one, 11.25% (466/4144) two, and 4.08% (169/4144) three or more chronic conditions. The most common chronic conditions were hypertension (763/4144, 18.41%), musculoskeletal conditions (385/4144, 9.29%), and cardiovascular diseases (376/4144, 9.07%). The mean BMI was 24.9 (SD 3.5) and 6.49% (269/4144) of the participants were classified as obese (BMI≥30).

In total, 87.02% (3606/4144) of participants reported adhering to doctor’s advice, 60.85% (2522/4144) were on a balanced diet, and 48.82% (2023/4144) were on a low fat diet. Furthermore, 38.95% (1614/4144) engaged in physical activity on a regular basis, and 28.50% (1181/4144) smoked daily. Average health-related quality of life was 3.9 out of 5 (SD 0.6) and mean health literacy was 33.5 out of 50 (SD 7.4).

The majority of the sample (2538/4144, 61.25%) reported using a smartphone and 20.53% (521/2538) of smartphone users also reported using health apps.

**Table 1:** Sample characteristics by smartphone and health app use.

Item		Total sample	Total sample		Smartphone users	
			No Smartphone^a^ use	Smartphone use	No health app use	Health app use
		100.00% (4144/4144)	38.75% (1606/4144)	61.25% (2538/4144)	79.47% (2017/2538)	20.53% (521/2538)
		N=4144	n=1606	n=2538	n=2017	n=521
**Female (vs male), n (%)**		2112 (50.97)	859 (53.49)	1253 (49.37)	984 (48.79)	269 (51.6)
**Age in years (SD^b^** **)**		57 (14)	68 (11)	50 (10)	51 (10)	47.9 (10)
**Educational level (ISCED^c^** **), n (%)**						
	No or basic qualification	534 (12.89)	229 (14.26)	305 (12.02)	244 (12.10)	61 (11.7)
	Vocational qualification	2858 (68.97)	1144 (71.23)	1714 (67.53)	1373 (68.07)	341 (65.5)
	University degree	752 (18.15)	233 (16.06)	519 (20.45)	400 (19.83)	119 (22.8)
**Occupational status, n (%)**						
	Working full-time	2224 (53.67)	309 (19.24)	1915 (75.45)	1503 (74.52)	412 (79.1)
	Working part-time	434 (10.47)	135 (8.41)	299 (11.78)	245 (12.17)	54 (10.4)
	Not working	198 (4.78)	81 (5.04)	117 (4.61)	92 (4.56)	25 (4.8)
	Retired	1287 (31.06)	1081 (67.31)	206 (8.12)	177 (9)	29 (5.6)
	In school	1 (0.02)	0 (0.00)	1 (0.04)	0 (0.00)	1 (0.20
**Monthly post-tax household income^d^** **, n (%)**							
	Low	1342 (32.38)	801 (49.88)	541 (21.32)	423 (20.97)	118 (22.6)
	Medium	1930 (46.57)	523 (32.57)	1407 (55.44)	1125 (55.78)	282 (54.1)
	High	290 (7.00)	40 (2.49)	250 (9.85)	201 (9.97)	49 (9.4)
	No answer	582 (14.04)	242 (15.07)	340 (13.40)	268 (13.29)	72 (13.8)
**First language, n (%)**						
	German	3773 (91.05)	1499 (93.34)	2274 (89.60)	1826 (90.53)	448 (86.0)
	Other	371 (8.95)	107 (6.66)	264 (10.40)	191 (9.47)	73 (14.0)
**Chronic conditions, n (%)**						
	Cardiovascular disease	376 (9.07)	275 (17.12)	101 (3.98)	81 (4.02)	20 (3.8)
	Cancer	79 (1.91)	49 (3.05)	30 (1.18)	24 (1.19)	6 (1.2)
	Respiratory diseases	232 (5.60)	117 (7.29)	115 (4.53)	94 (4.66)	21 (4.0)
	Musculoskeletal system conditions	385 (9.29)	277 (17.25)	108 (4.26)	84 (4.16)	24 (4.6)
	Depression	128 (3.09)	43 (2.68)	85 (3.35)	62 (3.07)	23 (4.4)
	Chronic pain	310 (7.48)	180 (11.21)	130 (5.12)	103 (5.11)	27 (5.2)
	Diabetes	361 (8.71)	201 (12.52)	160 (6.30)	117 (5.80)	43 (8.3)
	Hypertension	763 (18.41)	416 (25.90)	347 (13.67)	266 (13.19)	81 (15.5)
	Obesity	269 (6.49)	115 (7.16)	154 (6.07)	111 (5.50)	43 (8.3)
**Multiple chronic conditions, n (%)**						
	None	2231 (53.84)	553 (34.43)	1577 (62.14)	1270 (62.96)	307 (58.9)
	One	1278 (30.84)	599 (37.30)	684 (26.95)	546 (27.07)	138 (26.5)
	Two	466 (11.25)	300 (18.68)	213 (8.39)	153 (7.59)	60 (11.5)
	Three or more	169 (4.08)	154 (9.59)	64 (2.52)	48 (2.38)	16 (3.1)
**Health behaviors, n (%)**						
	Smoking	1181 (28.50)	364 (22.67)	817 (32.19)	658 (32.62)	159 (30.5)
	Physical activity	1614 (38.95)	468 (29.14)	1146 (45.15)	863 (42.79)	283 (54.3)
	Balanced diet	2522 (60.86)	992 (61.77)	1530 (60.28)	1189 (58.95)	341 (65.5)
	Low fat diet	2023 (48.82)	846 (52.68)	1177 (46.38)	895 (44.37)	282 (54.1)
	Adherence to doctor’s advice	3606 (87.01)	1338 (83.31)	2268 (89.36)	1803 (89.39)	465 (89.3)
	BMI^e^ (SD)	24.9 (3.5)	25.1 (3.7)	24.9 (3.5)	24.8 (3.3)	25.0 (3.9)
	Health-related quality of life (SD)	3.9 (0.6)	3.8 (0.6)	4.1 (0.6)	4.1 (0.6)	4.0 (0.6)
	Health literacy (SD)	33.5 (7.4)	33.1 (7.8)	33.0 (6.7)	34.9 (6.7)	35.7 (6.8)
**Behaviors targeted by the apps, n (%)**						
	Smoking cessation	232 (5.60)	0 (0.00)	232 (9.14)	0 (0.00)	232 (44.5)
	Regular physical activity	89 (2.15)	0 (0.00)	89 (3.51)	0 (0.00)	89 (17.1)
	Healthy diet	201 (4.85)	0 (0.00)	201 (7.92)	0 (0.00)	201 (38.6) (201/521)
	Weight loss	121 (2.92)	0 (0.00)	121 (4.77)	0 (0.00)	121 (23.2)
	Medication adherence	49 (1.18)	0 (0.00)	49 (1.93)	0 (0.00)	49 (9.4)
	Blood pressure control	36 (0.87)	0 (0.00)	36 (1.42)	0 (0.00)	36 (6.9)
	Blood sugar control	13 (0.31)	0 (0.00)	13 (0.51)	0 (0.00)	13 (2.5)
**App characteristics, n (%)**						
	Providing information	175 (4.22)	0 (0.00)	175 (6.90)	0 (0.00)	175 (33.6)
	Prompting motivation	179 (4.32)	0 (0.00)	179 (7.05)	0 (0.00)	179 (34.4)
	Planning	264 (6.37)	0 (0.00)	264 (10.40)	0 (0.00)	264 (50.7)
	Reminding	188 (4.54)	0 (0.00)	188 (7.41)	0 (0.00)	188 (36.1)
	Feedback or monitoring	94 (2.27)	0 (0.00)	94 (3.70)	0 (0.00)	94 (18.0)

^a^Smartphone refers to all kinds of Internet-compatible cell phones.

^b^SD: standard deviation.

^c^ISCED: International Standard Classification of Education.

^d^Post-tax household income: Low <€2100, moderate €2100-3600, high >€3600 (1 Euro=1.1 US dollar; October 6, 2016).

^e^BMI: body mass index.

### What Factors Are Associated With Smartphone Use?

Results from a binary logistic regression revealed that smartphone users were significantly younger (OR 0.92, *P*<.001) and were more likely to have a university degree (OR 1.69, *P*=.003). Furthermore, they were more likely to use the Internet for research about health issues (OR 3.24, *P*<.001) compared with non-smartphone users (see Table 2). Employment was associated with smartphone use, that is, individuals reporting to work part-time (OR 0.60, *P*<.001), not to work (OR 0.48, *P*<.001), and to be retired (OR 0.23, *P*<.001) were less likely to use smartphones compared with full-time workers. Participants who had a university degree were more likely to own a smartphone compared with people without or with basic qualification (OR 1.69, *P*=.003). Smartphone users were also more engaged in physical activity (OR 1.26, *P*=.02) but they were less likely to follow a low fat diet (OR 0.67, *P*<.001). Higher health-related quality of life was associated with smartphone use (OR 1.24, *P*=.03) and there was an association between health literacy (OR 1.05, *P*<.001) and smartphone use. Smartphone use was not associated with sex; mother tongue; chronic conditions; or the health behaviors smoking, balanced diet, and adherence.

**Table 2:** Multivariate associations with smartphone and health app use.

Item		Smartphone^a^ use			Health app use		
		(N=4144)			(n=2538)		
		Odds ratio	95% CI	*P*	Odds ratio	95% CI	*P*
**Intercept**		21.74^b^		<.001	0.47		0.19
**Female (vs male)**		0.92	0.76-1.1	0.37	1.15	0.94-1.42	0.18
**Age**		0.92^b^	0.91-0.93	<.001	0.97^b^	0.96-0.98	<.001
**Mother tongue (other vs German)**		1.18	0.85-1.64	0.32	1.5	1.11-2.02	0.008
**Internet use**		3.24^b^	2.6-4.03	<.001	1.68^b^	1.37-2.06	<.001
**Employment**							
	Working full-time	Reference^c^			Reference		
	Working part-time	0.60^b^	0.46-0.79	<.001	0.82	0.89-1.15	0.25
	Not working	0.48^b^	0.33-0.7	<.001	0.99	0.61-1.62	0.98
	Retired	0.23^b^	0.17-0.3	<.001	1.1	0.67-1.81	0.71
**Education**							
	No or basic qualification	Reference			Reference		
	Vocational qualification	1.04	0.8-1.35	0.79	0.89	0.65-1.23	0.49
	University degree	1.69	1.2-2.37	0.003	1.04	0.72-1.51	0.84
**Multiple chronic conditions**							
	None	Reference			Reference		
	One	1.02	0.83-1.26	0.85	1.34	1.04-1.72	0.02
	Two	0.97	0.72-1.3	0.83	2.36^b^	1.63-3.42	<.001
	Three or more	0.78	0.49-1.25	0.3	2	1.07-3.75	0.03
**Health behaviors**							
	Smoking	1.11	0.9-1.35	0.35	0.98	0.78-1.22	0.84
	Physical activity	1.26	1.03-1.52	0.02	1.38	1.11-1.72	0.003
	Balanced diet	1.03	0.85-1.26	0.75	1.06	0.84-1.34	0.63
	Low fat diet	0.67^b^	0.55-0.81	<.001	1.33	1.06-1.66	0.01
	Adherence (doctor’s advice)	0.96	0.72-1.27	0.76	0.81	0.57-1.14	0.23
**Health-related quality of life**		1.24	1.02-1.5	0.03	0.94	0.76-1.16	0.55
**Health literacy**		1.05^b^	1.04-1.07	<.001	1.02	1-1.03	0.04

^a^Smartphone refers to all kinds of Internet-compatible cell phones.

^b^Still significant after correction for multiplicity.

^c^Reference: reference category set to 1.

### Which Factors Are Associated With Health App Use?

Out of all smartphone users, 20.53% (n=521/2538) reported using health apps. Health app users were significantly younger (OR 0.97, *P*<.001) and used the Internet for research about health issues (OR 1.68, *P*<.001) more often than those not using health apps (see Table 2). Participants who had another language than German as mother tongue were more likely to use health apps (OR 1.50, *P*=.008). Individuals reporting chronic conditions were more likely to use health apps: individuals with one (OR 1.34, *P*=.02), two (OR 2.36, *P*<.001), and three or more chronic conditions (OR 2.00, *P*=.03) were more likely to use a health app compared with individuals without chronic conditions. Regarding health behaviors, a significant association with health app use was only found for physical activity (OR 1.38, *P*=.003) and low fat diet (OR 1.33, *P*=.01). Finally, health app users were more health literate than nonusers (OR 1.02, *P*=.04). Health app use was not associated with sex, employment, education, smoking, balanced diet, and adherence to doctor’s advice; and health-related quality of life.

### Which App Characteristics Do the Different Health Apps Include?

Health app users reported using apps to support changes in smoking cessation (232/521, 44.5%), healthy diet (201/521, 38.6%), weight loss (121/521, 23.2%), physical activity (89/521, 17.08%), medication adherence (49/521, 9.4%), blood pressure control (36/521, 6.9%), and blood sugar control (13/521, 2.5%; see Table 1). The most common app characteristic was planning (264/521, 50.7%), followed by reminding (188/521, 36.08%), prompting motivation (179/521, 34.4%), providing information (175/521, 33.6%), and feedback or monitoring (94/521, 18.0%).

### Which Behaviors Targeted by the Apps Are Associated With Actual Behavior?

Multimedia Appendix 1 shows associations of behaviors targeted by the apps and actual behavior. There are five separate analyses. All app users were included in each analysis (n=521). Covariates per the model are noted down the column on the left side, which is sex, age, behaviors targeted by the apps, and app characteristics.

Participants who were smokers were more likely to use health apps for physical activity (OR 14.44, *P*=.001; see Multimedia Appendix 1). Conversely, participants who reported to engage in regular physical activity were less likely to use apps targeting physical activity (OR 0.54, *P*=.03) and weight loss (OR 0.48, *P*=.003) and were more likely to use apps targeting smoking cessation (OR 3.01, *P*<.001) and healthy diet (OR 1.65, *P*=.02) compared with inactive participants. Participants who were engaged in physical activity were also significantly younger (OR 0.97, *P*=.001) than inactive ones. Individuals following a low fat diet were more likely to use apps on smoking cessation (OR 2.34, *P*<.001) and healthy diet (OR 1.83, *P*=.007), but were less likely to use apps targeting weight loss (OR 0.31, *P*<.001). Individuals engaging in a balanced diet were using apps for smoking cessation (OR 2.16, *P*=.001) and healthy diet (OR 1.97, *P*=.003) more often, but used apps for weight loss (OR 0.31, *P*<.001) less often. Participants who reported adherence to doctor’s advice were more likely to use apps on smoking cessation (OR 3.07, *P*=.005). There were no other associations between behaviors targeted by the apps and actual behavior beyond the reported findings.

### Which App Characteristics Are Associated With Actual Behavior?

Multimedia Appendix 1 shows associations of behaviors targeted by the apps and actual behavior. There are five separate analyses. All app users were included in each analysis (n=521). Covariates per the model are noted down the column on the left side, that is sex, age, behaviors targeted by the apps, and app characteristics.

Participants using health apps including the app characteristics, planning (OR 1.51, *P*=.04) and feedback or monitoring (OR 2.15, *P*=.007), were more likely to engage in physical activity (see Multimedia Appendix 1). Moreover, people who reported to use apps including the app characteristic feedback or monitoring were more likely to adhere to doctoral advice (OR 5.94, *P*=.02). There were no other associations between app characteristics and actual behavior beyond the reported findings.

## Discussion

### Principal Findings

Two-thirds of the participants owned a smartphone, but only one in five reported using apps for health-related purposes. Those using smartphones tended to differ in their demographic profile compared with nonusers. They tended to be younger, more health literate, had a higher socioeconomic status, and felt better about their life. Importantly, those who used health apps appeared to be different from smartphone owners who did not use health apps. Health app users were younger, less likely to be native German speakers, more likely to conduct health-related Internet searches, more likely to suffer from chronic conditions, and more likely to follow health behaviors. Although using a health app may reveal an interest in health and certain health behaviors, the apps that people were using were not necessarily reflective of the health behaviors they were performing. Hence, health app use may reflect a user’s motivation to change health behaviors. Finally, we found weak associations of health behaviors and health app characteristics, with planning and self-monitoring being the only significant exceptions.

### Smartphone Use

The extent of smartphone use found in our study was comparable with the findings of the 2015 US Pew Research Center Survey [23], where 64% of the participants were using a smartphone, compared with 61.25% (2538/4144) in our sample. A 2012 Pew Research Center Survey found that 45% of the US adults owned a smartphone [24]. In a recent longitudinal survey, Levine et al [10] found that older people with a mean age of 75 years used digital health at low rates. There were modest increases from 2011 through 2014. This underlines the rising importance of mobile technology in people’s lives. The 2015 Pew Survey also identified age as a correlates for smartphone use, which was confirmed in this study: younger people were more engaged [23]. Furthermore, our findings contribute to previous findings on literacy-related disparities in access to mobile technologies by revealing an association between smartphone use and health literacy [9].

We contributed to the evidence of correlates of smartphone use with health-related quality of life, Internet research behavior, physical activity, and low fat diet. The relationship between health-related quality of life and smartphone use may be explained by socioeconomic variables. People with higher education and higher incomes score higher in quality of life [25]. Indeed, we found a university degree and a full-time employment to be associated with smartphone use. Further research on this interplay is needed. Smartphones offer direct and easy access to the Internet, which may be a possible explanation for the correlation between Internet research behavior and smartphone use. This finding underlines the importance of the Internet and mobile technologies for health issues.

### Health App Use

Regarding health app use, the 2012 Pew Survey found that 19% of the mobile phone users took advantage of health apps, consistent with our finding of 20.53% (521/2538) [24]. In both studies, 4 out of 5 participants were not using health apps. Results from a recent study by Krebs and Duncan [7] revealed a health app use of nearly 60%. Krebs and Duncan investigated a younger population with a mean age of 40 years (mean age of our sample was 57 years) which may be a possible explanation for the difference. Nonetheless, this underlines the potential of health apps in the future, as they seem to be very popular among young people; yet also older people are increasingly engaged in mobile technologies [10,23].

We found native German speakers to be less likely to use health apps compared with nonnative speakers. This finding is consistent with Krebs and Duncan, who found in their survey among a US sample that hispanics were more likely to use health apps. Greater use by minorities in each country could be due to difficulties accessing the health care system as well as greater use of Internet on smartphones versus home connections.

We found that the association of health app use, age, and health-literacy in our survey was in accordance with results from Krebs and Duncan [7] and Bailey et al [9], who found age and literacy-related disparities in the use of mobile health apps. The role of age in the use of health apps highlights that the relevance of new potential ways of supporting health topics is growing in the future. However, app developers should not forget about older people, especially because health issues become increasingly important in later years [26]. Tailored theory-based health app interventions may be a way to reach people from all ages, and app developers should take the needs of older people and people with low health literacy into account to further decrease the “digital gap” between users and nonusers [2].

There is a need for mobile solutions for disease management. Our findings indicate that people suffering from chronic conditions were more likely to use health apps. We found this correlation not only when we were looking at smartphone users (n=2538) but also when we were investigating the whole sample (N=4144). Another finding from this study is the correlation between physical activity and health app use, that is, people who exercise are more likely to use health apps. Both findings indicate that people use mobile technologies that help them to manage diseases and certain health behaviors.

Looking at the behaviors targeted by the apps, the 2012 Pew Survey identified exercise, diet, and weight loss apps as the most popular behaviors [24]. In this study, we found smoking cessation, healthy diet, and weight loss to be the most popular behaviors targeted by the apps. Considering that cardiovascular diseases are the most common cause of death in western countries, this is an encouraging finding [27].

### Behaviors Targeted by the Apps and Characteristics of the Apps

These analyses were conducted to explore the correlations between health behaviors targeted by the apps, and health app characteristics and actual health behavior, rather than to strictly test hypotheses. Future research should establish specific hypotheses based on our results. In this study, health app use was seen as motivation to change or maintain health behaviors using mobile technologies. One-fifth of the smartphone users reported use of health apps, which may reflect an interest in health and health behavior change. Conversely, using health apps was not necessarily related to an active engagement in the respective health behavior. It appears that the apps which people have on their smartphones indicate what they want to change, not their actual behavior. Concerning correlations of health app characteristics with actual behavior there were associations between planning and physical activity, between feedback or monitoring and physical activity, and between feedback or monitoring and adherence to doctor’s advice. These findings relate to other studies that found self-monitoring to be associated with health behaviors—although our findings were limited to certain health behaviors [28].

### Strengths and Limitations

A strength of this study was the large nationwide sample. It was a stratified sample by sex, age, German federal state, and education to increase the representativeness. Moreover, we provided an overview about the extent of smartphone and app use. Finally, we supplied novel information, contributing to the research field, by including health-related quality of life, health literacy, multiple chronic conditions, and health behaviors as well as information about health app characteristics aimed at changing health behaviors.

Our research was limited by the design of the survey. Due to the cross-sectional character of the survey, changes could not be examined, including associations of behaviors targeted by the apps and app characteristics with actual behavior. As the findings suggest, smartphones and app use were more common among younger respondents; however, the survey did not include people aged below 35 years, as our focus was individuals with and without chronic conditions. Income could not be used in multivariate predictions because a critical number of participants (582/4144, 14.04%) did not answer that question. Previous studies found app use to be associated with higher income [7]. Another aspect is that the nature of the survey questions limited the conclusions that could be drawn from the results. For example, BMI was calculated based on self-reported weight and height which is a possible source of bias. Smoking was defined as daily smoking which overlooks the complexity of smoking behavior, for example, social smoking. This limits the conclusions that can be drawn from the finding that smoking status was not associated with the use of apps for smoking cessation.

Furthermore, a large number of analyses were conducted increasing the probability of type I errors (ie, stating an effect when none was present). However, our study has an explorative character. Nonetheless, in addition to the uncorrected results, we decided to report the multiplicity corrected results following a recommendation by Streiner [22], who discussed arguments for and against a correction of multiplicity.

Namely, our ambition is to provide a huge basis that can be further examined in future research.

### Implications for Further Research, Policy, and Practice

Our findings have shown that two-thirds of the participants aged above 35 years used a smartphone, but of these only 1 in 5 participants has been using health apps. Thus, when apps are designed for public health, many people cannot be reached because they either do not have a smartphone or they do not use it for health reasons. Furthermore, lower levels of health literacy and age appropriateness need to be taken into account when designing health apps. Special regard for people with multiple chronic conditions is needed. Tailoring the training regime to the special needs of chronically ill people have not yet been implemented in many health and lifestyle apps. Health apps may play a major role for future health participation of individuals as well as for their autonomy and health literacy.

We gained insight into the relationship between app characteristics and actual behavior. For future research, the effects of health app use on health behaviors should be analyzed by applying longitudinal or experimental research designs. An important point, which should be investigated, is the association between the presence or absence of chronic conditions and the use of specific health apps or app characteristics. Future research should investigate additional behaviors targeted by apps such as sleep tracking or managing doctoral appointments as our questions constrained the survey results to certain behaviors based on the review by Riley et al [2].

The influence of health app use on health-related quality of life, health behaviors, and disease management should be investigated in greater detail. More research on effective app characteristics is needed. Information on the effectiveness of health apps based on evidence from rigorous research designs should be provided for users. This would increase the transparency in the market, given the huge number of apps available. Not only governmental guidelines and regulations, but also WHO recommendations could help people choose effective and appropriate apps [29]. A first attempt in this direction is the MEDDEV Guideline 2.1/6 for the European market and a guideline from the Food and Drug Administration (FDA) for the US market [30,31]. Although these are not legally binding, they offer an orientation for developers and consumers of mobile health apps.

We found age- and literacy-related disparities in the use of mobile technologies. Thus, app developers and researchers should take the needs of older people with multiple chronic conditions and people with low health literacy, for example, by providing tailored health apps tested in intervention studies, into account [7,9]. Likewise, campaigns should be launched aimed at training older adults in mobile technologies and enhancing the health literacy of the population to decrease inequalities resulting from technological progress.

## Appendix

Multimedia Appendix 1

## Abbreviations

BMI: body mass index
CAPI: computer-assisted personal interviews
EUROHIS-QOL: European Health Interview Survey-Quality of Life
FDA: Food and Drug Administration
HLS-EU-Q: European Health Literacy Survey Questionnaire
ISCED: International Standard Classification of Education
WHO: World Health Organization
