# Supplementary material for: Using Smartphones and Health Apps to Change and Manage Health Behaviors: A Population-Based Survey
Source: J Med Internet Res. 2017 Apr 5;19(4):e101. doi: 10.2196/jmir.6838 (PMC5399221; doi:10.2196/jmir.6838)
Supplement: Multimedia Appendix 1 [file jmir_v19i4e101_app1.pdf]

Table 3. Multivariate associations of behaviors targeted by the apps and app characteristics with smoking among health app users (n=521).

**Covariates**		**OR[a] (95% CI)**	***P***
	Intercept	0.28	.40
	Female (vs male)	0.83 (0.53-1.29)	.41
	Age in years	1.01 (0.99-1.03)	.52
**Behaviors targeted by the apps**			
	Regular physical activity	14.44 (7.64-27.29)	.001
	Smoking cessation	0.93 (0.57-1.52)	.77
	Healthy diet	0.67 (0.41-1.09)	.11
	Weight loss	1.26 (0.74-2.15)	.40
	Medication adherence	2.03 (1.00-4.12)	.05
	Blood pressure control	0.67 (0.28-1.61)	.37
	Blood sugar control	1.01 (0.26-3.91)	.99
**App characteristics**			
	Providing information	0.96 (0.59-1.56)	.87
	Prompting motivation	0.97 (0.60-1.58)	.90
	Planning	0.67 (0.43-1.05)	.08
	Reminding	1.13 (0.71-1.81)	.60
	Feedback or monitoring	0.55 (0.29-1.04)	.07

[a]OR: odds ratio.

Table 4. Multivariate associations of behaviors targeted by the apps and app characteristics with physical activity among health app users (n=521).

**Covariates**		**OR[a] (95% CI)**	***P***
	Intercept	3.33	.03
	Female (vs male)	0.88 (0.59-1.30)	.52
	Age in years	0.97 (0.95-0.99)	.001
**Behaviors targeted by the apps**			
	Regular physical activity	0.54 (0.31-0.95)	.03
	Smoking cessation	3.01 (1.96-4.65)	<.001[b]
	Healthy diet	1.65 (1.07-2.55)	.02
	Weight loss	0.48 (0.30-0.78)	.003
	Medication adherence	1.05 (0.54-	.90

		2.04)	
	Blood pressure control	0.71 (0.33-1.53)	.39
	Blood sugar control	1.99 (0.57-6.97)	.28
**App characteristics**			
	Providing information	0.91 (0.60-1.39)	.67
	Prompting motivation	0.87 (0.56-1.33)	.51
	Planning	1.51 (1.02-2.24)	.04
	Reminding	1.12 (0.73-1.71)	.60
	Feedback or monitoring	2.15 (1.23-3.78)	.007

[a]OR: odds ratio.
[b]still significant after correction for multiplicity

Table 5. Multivariate associations of behaviors targeted by the apps and app characteristics with low fat diet among health app users (n=521).

**Covariates**		**OR[a] (95% CI)**	***P***
	Intercept	1.09	.88
	Female (vs male)	1.16 (0.79-1.71)	.44
	Age in years	0.99 (9.97-1.01)	.14
**Behaviors targeted by the apps**			
	Regular physical activity	0.81 (0.46-1.40)	.45
	Smoking cessation	2.34 (1.52-3.60)	<.001[b]
	Healthy diet	1.83 (1.18-1.83)	.007
	Weight loss	0.31 (0.19-0.50)	<.001[b]
	Medication adherence	1.14 (0.58-2.23)	.71
	Blood pressure control	1.53 (0.73-3.23)	.26
	Blood sugar control	1.98 (0.55-7.11)	.30
**App characteristics**			
	Providing information	1.27 (0.83-	.27

		1.92)	
	Prompting motivation	1.17 (0.77-1.77)	.48
	Planning	1.45 (0.99-2.14)	.06
	Reminding	0.93 (0.61-1.41)	.73
	Feedback or monitoring	1.66 (0.97-2.84)	.06

[a]OR: odds ratio.
[b]still significant after correction for multiplicity

Table 6. Multivariate associations of behaviors targeted by the apps and app characteristics with balanced diet among health app users (n=521).

**Covariates**		**OR[a] (95% CI)**	***P***
	Intercept	1.54	.45
	Female (vs male)	1.02 (0.69-1.52)	.91
	Age in years	0.99 (0.97-1.01)	.44
**Behaviors targeted by the apps**			
	Regular physical activity	1.11 (0.63-1.95)	.73
	Smoking cessation	2.16 (1.38-3.39)	.001
	Healthy diet	1.97 (1.26-3.09)	.003
	Weight loss	0.32 (0.20-0.52)	<.001[b]
	Medication adherence	1.52 (0.75-3.10)	.25
	Blood pressure control	0.72 (0.34-1.50)	.38
	Blood sugar control	1.31 (0.36-4.77)	.69
**App characteristics**			
	Providing information	0.88 (0.57-1.35)	.56
	Prompting motivation	1.01 (0.66-1.56)	.96
	Planning	1.35 (0.91-2.02)	.14
	Reminding	1.27 (0.82-	.28

		1.95)	
	Feedback or monitoring	1.55 (0.88-2.73)	.13

[a]OR: odds ratio.
[b]still significant after correction for multiplicity

Table 7. Multivariate associations of behaviors targeted by the apps and app characteristics with adherence to doctor's advice among health app users (n=521).

**Covariates**		**OR[a] (95% CI)**	***P***
	Intercept	6.73	.02
	Female (vs male)	0.83 (0.46-1.49)	.53
	Age in years	0.99 (0.96-1.02)	.43
**Behaviors targeted by the apps**			
	Regular physical activity	1.72 (0.67-4.42)	.26
	Smoking cessation	3.07 (1.40-6.72)	.005
	Healthy diet	1.76 (0.84-3.65)	.13
	Weight loss	1.02 (0.49-2.15)	.96
	Medication adherence	0.81 (0.31-2.15)	.68
	Blood pressure control	1.78 (0.49-6.43)	.38
	Blood sugar control	1.62 (0.18-14.63)	.67
**App characteristics**			
	Providing information	1.05 (0.56-1.98)	.88
	Prompting motivation	0.60 (0.32-1.13)	.11
	Planning	1.29 (0.72-2.34)	.40
	Reminding	1.09 (0.57-2.07)	.80
	Feedback or monitoring	5.94 (1.37-25.66)	.02

[a]OR: odds ratio.
